# Psychiatric Burden and Suicide Risk in Von Hippel-Lindau Syndrome: A Case-based Psychosocial Analysis

**DOI:** 10.1192/j.eurpsy.2026.11440

**Published:** 2026-07-31

**Authors:** N. Bouayed Abdelmoula, R. Rhaiem, N. Boukthir, B. Abdelmoula

**Affiliations:** LR23ES07 Genomics of Signalopathies at the service of Precision Medicine, Medical University of Sfax, Sfax, Tunisia

## Abstract

**Introduction:**

Von Hippel-Lindau syndrome (VHL) is a rare autosomal dominant disease caused by mutations in the VHL tumor suppressor gene, characterized by multi-organ vascular tumors. Genetic counseling plays an essential role in diagnosis, family screening, risk assessment and management.

**Objectives:**

To illustrate the complex clinical, genetic and psychiatric dimensions of VHL through a familial case, and highlight the importance of integrated psychiatric care in long-term management.

**Methods:**

We present a descriptive clinical case study of a patient carrying type 2B VHL, with detailed documentation of her medical, neurological, oncological, psychiatric and family history.

**Results:**

We report the case of a 50-year-old woman with type 2B VHL, who presented with progressive cerebellar syndrome at the age of 42, caused by multiple cerebellar and cervical spinal hemangioblastomas, and a history of recurrent right retinal angiomas. Over time, she developed a clear-cell multifocal renal cell carcinoma (left kidney), a right adrenal pheochromocytoma, multiple pancreatic cysts and a benign ovarian cyst. A partial thrombosis of the left renal vein revealed a bifocal recurrence of renal cell carcinoma, managed by targeted treatment. The family history revealed 11 siblings, 6 of whom were affected. Four died prematurely, three before the age of 21 and one at the age of 47, from mainly neurological complications related to VHL. Only the patient and her 48-year-old brother (also suffering from kidney damage) are currently alive, probably reflecting the progression of the age-related disease. The psychiatric burden has been considerable. The accumulation of neurological disabilities, oncological recurrences, physical exhaustion and pain caused by the loss of several brothers and sisters contributed to a major depressive episode, leading to a suicide attempt. This underlines the dimension of mental health that is often neglected in the clinical management of hereditary cancer syndromes.

**Conclusions:**

This case illustrates the multisystem and hereditary nature of VHL, as well as the chronic psychological stressors that affected individuals face. Beyond genetic counseling and tumor monitoring, comprehensive psychiatric evaluation and support should be considered essential elements of the management of VHL. Early identification and treatment of psychiatric comorbidities, in particular depression and suicidal ideation, can significantly improve outcomes and quality of life. Multidisciplinary approaches involving neurology, oncology, psychiatry, genetics and palliative care are essential to meet all the needs of patients with complex hereditary diseases.

**Disclosure of Interest:**

None Declared

